# Diffusion Modelling Reveals the Decision Making Processes Underlying Negative Judgement Bias in Rats

**DOI:** 10.1371/journal.pone.0152592

**Published:** 2016-03-29

**Authors:** Claire A. Hales, Emma S. J. Robinson, Conor J. Houghton

**Affiliations:** 1 School of Physiology and Pharmacology, Faculty of Biomedical Sciences, University of Bristol, Bristol, BS8 1TD, United Kingdom; 2 Department of Computer Science, Faculty of Engineering, University of Bristol, Bristol, BS8 1UB, United Kingdom; Radboud University Medical Centre, NETHERLANDS

## Abstract

Human decision making is modified by emotional state. Rodents exhibit similar biases during interpretation of ambiguous cues that can be altered by affective state manipulations. In this study, the impact of negative affective state on judgement bias in rats was measured using an ambiguous-cue interpretation task. Acute treatment with an anxiogenic drug (FG7142), and chronic restraint stress and social isolation both induced a bias towards more negative interpretation of the ambiguous cue. The diffusion model was fit to behavioural data to allow further analysis of the underlying decision making processes. To uncover the way in which parameters vary together in relation to affective state manipulations, independent component analysis was conducted on rate of information accumulation and distances to decision threshold parameters for control data. Results from this analysis were applied to parameters from negative affective state manipulations. These projected components were compared to control components to reveal the changes in decision making processes that are due to affective state manipulations. Negative affective bias in rodents induced by either FG7142 or chronic stress is due to a combination of more negative interpretation of the ambiguous cue, reduced anticipation of the high reward and increased anticipation of the low reward.

## Introduction

Affective states alter cognitive processes. The measurable consequences of these alterations are referred to as affective biases [1–3]. They have most often been studied in relation to emotional disorders including anxiety and depression, where negative biases in attention, memory, perception of emotional stimuli and interpretation of ambiguous information are linked to negative emotional states (see [4–7] for in-depth reviews). It has been suggested that these biases may be important in the development, maintenance and treatment of both anxiety and depression [8–13].

Different affective states have been shown to be associated with positive and negative judgement biases in many different species of animals (see [14, 15] for recent reviews) and in humans [16–19]. This association was first demonstrated by Harding et al. [20] in rats. In this study, induction of putative negative affective state was associated with a negative judgement bias in an ambiguous-cue interpretation task where rats were required to respond for reward and withhold responding to avoid punishment. Since this initial study, tasks involving an operant go/go format have been developed. These avoid confounds associated with motivational changes that may occur with go/no-go formats. In these reward-punishment (R-P) tasks, similar negative judgement biases have been observed following pharmacological manipulations [21–23] and chronic stress procedures [24, 25]. These behavioural readouts may provide a method to assess animal emotion, providing a framework for investigating the links between affective state and cognition in a rodent model [26].

Mathematical models, such as the diffusion model [27, 28], can be used to model the cognitive processes that are involved in two-choice decisions. The diffusion model has been extensively applied to human decision making tasks, including those studying emotional processing [29–31] and bias [32] to provide insight into the underlying cognitive processes that become dysfunctional in mood disorders (see [33] for a review). However, this computational approach has not been applied previously to rodent two-choice response time (RT) behavioural data. The rodent ambiguous-cue interpretation task provides this type of data, and enables measurement of judgement bias following direct manipulation of affective state. Decision making in this task can be investigated using a diffusion model-based approach, as model parameters can be related intuitively to our understanding of the underlying neural processes. This cannot be explored through traditional analyses using behavioural data alone.

**Fig 1:**
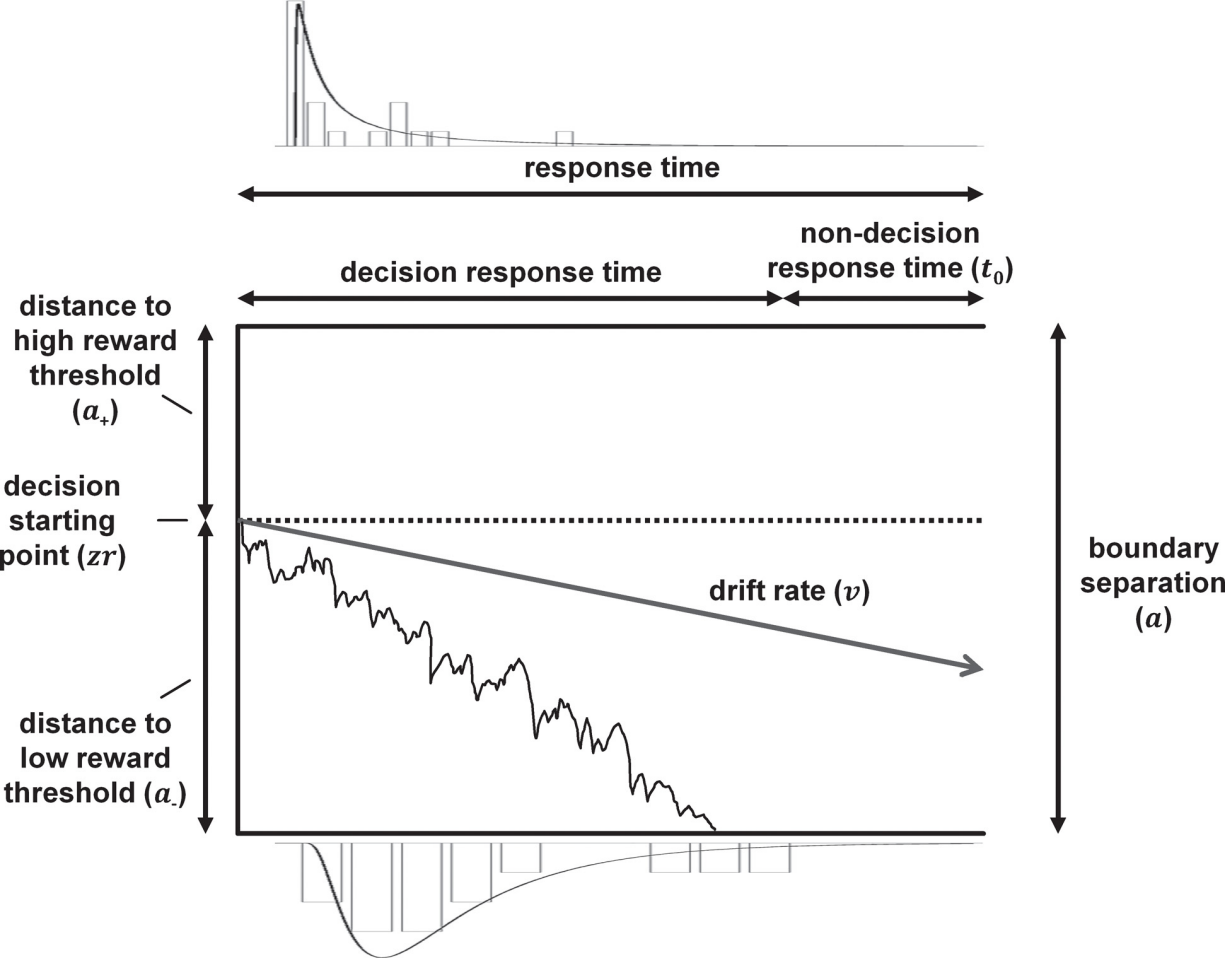
Diagrammatic representation of the diffusion model for a simple two-choice response time task. The vertical axis shows boundary separation (*a*): the distance between the two decision thresholds. The upper boundary corresponds to a decision to press the high reward lever, while the lower boundary corresponds to a decision to press the low reward lever. The decision is made through information accumulation, which begins from a starting point (*zr*) located somewhere between the two boundaries. *a*_+_ is the distance between the decision starting point and the upper boundary, while *a*_-_ is the distance between the starting point and lower boundary. *a*_*+*_ *= a*_*-*_ would mean the decision starting point is exactly halfway between the two boundaries (signifying no bias). The horizontal axis is the total response time (RT), made up of the decision time plus the non-decision RT (*t*_*0*_). Representative RT distributions with behavioural data (bars) and RT curves used by the model to calculate parameters (black lines) for the midpoint tone are shown above and below the model for decisions cumulating in high and low reward lever presses respectively. The RT distributions are from a single rat on a single control session, which was chosen by selecting the data point with median goodness of fit *p*-value (*p* = 0.862) from all control data. The jagged line shows an example decision making process which is noisy, and has an average rate of information accumulation called the drift rate (*v*). The relative positions of boundaries, starting point and gradient of the drift rate are drawn scaled to the average parameter values for all control data for the midpoint tone.

The diffusion model [27, 28] (Fig 1) breaks down the decision making process into components which include the decision starting point, decision criteria (thresholds), quality of incoming information and the time taken for non-decision processes. During the decision process, information accumulates over time in random small steps. This begins from the decision starting point (*zr*) and ends once one of the two decision thresholds (each representing one of the two alternative choices) are reached. This accumulation process is modelled by a random-walk that is biased by the quality of incoming information. This bias, or drift rate (*v*) is disrupted by noise, which allows the model to account for a distribution of RTs, as well as errors. The decision starting point (representing response bias) and decision threshold parameters can be used to calculate distances from the starting point to both the upper (*a*_*+*_) and lower (*a*_*-*_) boundaries. The model also separates time taken to accumulate information from other non-decision processes such as time taken for perceptual encoding and to execute motor responses (*t*_*0*_). In addition, across-trial variability in the drift rate (*sv*), starting point (*szr*) and in the non-decision time component (*st*_*0*_), as well as differences in speed of response execution (*d*) can be accounted for in the model. The use of the diffusion model may be particularly advantageous in animal behavioural tasks, as the model incorporates full temporal profiles of behavioural responding. This means that more dimensions from the original data are taken into account than with traditional analyses of behavioural data, where typically either RTs or response outcomes are analysed. This provides a richer description of the data, and when combined with independent component analysis (ICA) reveals the underlying decision making components that are altered by negative affective state manipulations.

This study measured judgement bias following negative affective state manipulations using a reward-reward (R-R) operant go/go ambiguous-cue interpretation task. Rats were trained to discriminate between two distinct auditory tones and make a response on the appropriate lever to obtain either a high value reward (four pellets) or a low value reward (one pellet). Responding to a midpoint ambiguous tone was measured as an indicator of judgement bias following acute (restraint stress or administration of FG7142: a GABA_A_ receptor partial inverse agonist) and chronic (psychosocial stress) negative affective state manipulations. Behavioural data were fit to the diffusion model to investigate the processes involved in decision making. Parameters corresponding to rate of information accumulation and distances from decision starting point to boundaries were analysed conventionally and using ICA to probe the specific changes underlying the observed negative judgement bias.

## Materials and Methods

### Animals and apparatus

Sixteen male Lister hooded rats were used in the experiments (Harlan, UK). Rats weighed ~ 270 g at the start of training, and ~ 500 g at the start of experimental manipulations (see S1 Table for details of this period). Rats were kept on a 12 hour reverse lighting cycle (lights off at 0800 hours) and under temperature- and humidity-controlled conditions. Water was available *ad libitum* in the home cage, but rats were maintained at no less than 90% of their free-feeding body weight by restricting access to laboratory chow (LabDiet, PMI Nutrition International) to ~ 18 g per rat per day. All procedures were carried out under local institutional guidelines (approved by the University of Bristol Animal Welfare and Ethical Review Board) and in accordance with the UK Animals (Scientific Procedures) Act 1986. Rats were housed two per cage with environmental enrichment consisting of a red 3 mm Perspex house (30 x 10 x 17 cm), a cardboard tube and a cotton rope suspended across the cage lid to improve animal welfare. During experiments all efforts were made to minimise suffering, and at the end of experiments rats were euthanised by giving an overdose of sodium pentobarbitone. All behavioural testing was carried out between 0800 and 1800 hours. Behavioural experiments were carried out using standard rat operant chambers (Med Associates, Sandown Scientific, UK). For details of operant chamber configuration, see S1 File.

### Behavioural task

Rats were trained to associate one tone (2 or 8 kHz at 75 and 64 dB respectively; counterbalanced across rats, designated “high reward”) with a large food reward (four 45 mg reward pellets; Test Diet, Sandown Scientific, UK) and the other tone (8 or 2 kHz, the opposite of the high reward tone, designated “low reward”) with a small food reward (one 45 mg reward pellet) if they pressed the associated lever during the 20 s tone. For training stages and specific task details see S1 File and S1 Table. Baseline sessions consisted of 100 trials (50 high reward and 50 low reward tones presented pseudorandomly). Probe test sessions consisted of 120 trials: 40 high reward tones, 40 low reward tones, and 40 ambiguous midpoint tones made up of one of two tones (20 x 4,999 Hz and 20 x 5,001 Hz at 70 dB). These two midpoint tones are impossible to distinguish, but are required to enable random reinforcement of the midpoint tone. This is achieved through each of the ambiguous tones having the same outcome as the reference tone they were closer to. Responses to either of the two midpoint tones were analysed together. Random reinforcement was used so that rats could not learn a specific outcome for the midpoint tone, and to ensure continuing responding to that tone throughout sessions and across the experiments. Unlike in the R-P version of the task where one cue is associated with a lack of outcome (avoidance of punishment) [21–25], both reference tones are associated with the occurrence of an outcome (delivery of reward) in this R-R task. This means that over the repeated test sessions used in this study, rats would learn there is no outcome associated with the ambiguous tone if it was unrewarded, as has previously been shown in a similar reward-based judgement bias task in starlings that experienced multiple testing sessions [34].

Rats were considered trained when they responded with > 60% accuracy on both reference tones for three consecutive days on the final training stage (Stage 5, S1 Table), and were excluded from analyses if they failed to maintain > 60% accuracy during experiments. All rats (*n* = 16) completed discrimination training (Stage 4; S1 Table) and thirteen rats met criteria for reward magnitude training (Stage 5; S1 Table) before experimental manipulations began. Rats experienced at least one week re-baseline (five baseline sessions) between each experimental manipulation.

### Experiment 1: Acute induction of negative affective state

Two acute manipulations–a stress manipulation: acute restraint stress; and a pharmacological manipulation: acute administration of the anxiogenic drug FG7142 (a GABA_A_ receptor partial inverse agonist)–and were used to examine the effects of acute negative affective state manipulations on judgement bias. Twelve rats underwent both manipulations (four were excluded for consistently failing to meet task criteria in baseline sessions). Each manipulation followed a within-subject study design with fully counterbalanced treatments. Manipulations followed by probe test sessions were conducted on Tuesday and Friday, with baseline sessions on Monday and Thursday, and no testing on Wednesday or at the weekend. For restraint stress, rats were placed into a restraint tube placed for 15 min immediately prior to probe test sessions. The restraint tube was placed inside an operant chamber identical to the ones used for testing but located in another room so that induction of affective state change, and testing for this change, occurred in the same context. The control treatment for this manipulation was normal housing conditions. A further one rat did not meet task criteria during the control session and so was subsequently excluded from analysis of this manipulation. FG7142 (3.0, 5.0 mg/kg, purchased from Sigma-Aldrich, UK) or vehicle (10% dimethyl sulfoxide, 20% cremophor, 70% saline) was given via intraperitoneal injection using a low-stress, non-restrained technique [35] 30 min prior to testing with a dose volume of 1 ml/kg. Ten of the twelve rats that underwent this manipulation met criteria during the vehicle session to be included in the analysis. Drug doses were selected based on previous affective bias studies [36].

### Experiment 2: Chronic induction of negative affective state

A chronic stress manipulation—repeated restraint stress and social isolation (RS&SI)—was used to assess the effect of a longer-term negative affective state manipulation on judgement of the midpoint ambiguous tone. This procedure has previously been shown in our laboratory to reliably induce negative affective state [36, 37]. This study used a between-subjects study design, and was split into three parts: (1) a pre-stress week, (2) three weeks of testing with stress manipulation, and (3) three weeks of post-stress testing. Rats experienced baseline sessions on Monday, Wednesday and Thursday, with probe test sessions on Tuesday and Friday. Rats were split into two groups (control or RS&SI, *n* = 8 per group) based on performance during the pre-stress week. The two groups were matched for all analysed behavioural variables. RS&SI rats experienced isolation housing (in unenriched cages with paper partitions between to prevent visual contact with other rats) and daily repeated restraint stress (as for the acute manipulation) during the second part of the study, before being returned back to control housing with no restraint stress for the final three weeks. Restraint stress occurred immediately prior to both baseline and probe test sessions for the duration of the stress manipulation, therefore rats experienced daily (five days per week) repeated restraint stress. Control rats remained pair housed in standard enriched cages and did not experience any restraint stress. Three rats per group subsequently had to be excluded from analysis as they failed to meet task criteria.

### Modelling

The diffusion model was fit using fast-dm-30 [38–41]. Rather than simulating random repeated runs of the information accumulation process, fast-dm calculates predictive cumulative distribution functions (CDFs) for RTs, and then uses a partial differentiation equation solver to model the evolution of the probability distribution forward in time. Parameters are optimised by using an implementation of the Nelder-Mead method [42]. Further details about diffusion modelling using fast-dm can be found in Voss et al. [41]. Validation of model fit was carried on behavioural data from two probe test sessions conducted prior to experimental manipulations. Details of model fit validation can be found in S1 File. The parameter combination which produced the best model fit was selected and used to model the behavioural data from each probe tone session conducted during both experiments. Data from individual rats were modelled separately. Three parameters corresponding to relative starting point (*zr*), boundary separation (*a*) and drift rate (*v*) were fit to each tone using all trials for that tone within a session. These parameters were used to calculate two measures, which along with drift rate (*v*) were analysed for each experimental manipulation:

1. distance from the starting point to the upper boundary (*a*_*+*_):
  $$a_{+}\;=\;zr\;*\;a$$
2. distance from the starting point to the lower boundary (*a*_*-*_):
  $$a_{-}\;=\;a\;-\;a_{+}$$

Three other parameters (the variability in decision starting point: *szr*, the non-decision time: *t*_*0*_, and the difference in speed of response execution between the two responses: *d*) were fit using data from all three tones together (all trials within a session). The variability in drift rate (*sv*) and non-decision RT (*st*_*0*_) were set to 0. In the model, the upper boundary represents a decision to press the high reward lever, while the lower boundary represents a decision to press the low reward lever. Therefore, for each tone presentation, data entered into the model were tone, response latency and which lever (high or low reward) the response was made on (corresponding to whether the decision process reached the upper or lower boundary). For further details see S1 File.

### Independent Component Analysis

ICA was conducted on control diffusion model data from probe test sessions from all experiments (*n* = 101 data points). Three measures from diffusion model parameters (distance to the upper boundary: *a*_*+*_, distance to the lower boundary: *a*_*-*_, and drift rate: *v*) were used for the midpoint tone only. To ensure all measures were in equivalent units, the variance of *v* was normalised to the variance of *a*_*+*_ and *a*_*-*_ following mean centring. *a*_*+*_, *a*_*-*_ and normalised *v* were then input into the FastICA package [43, 44] in Matlab R2014a (8.3.0.532) (The Mathworks, Inc., USA). The separation matrix (***W***) was used to project *a*_*+*_, *a*_*-*_ and *v* from negative affective state manipulation data (*n* = 91 data points) into the independent component space found from ICA on control data. Independent component scores for each data point from each negative affective state manipulation were then compared to the scores for all control data from each manipulation grouped together. Diagrams representing the direction of change of diffusion model parameters for each independent component were constructed to scale using the average values for diffusion model parameters from all data (control and negative affective state). The directions and magnitude of change were determined from the unit vectors of the separating matrix (***W***). These unitised values are reported in the Results section.

### Statistical Analysis

The behavioural test measures analysed and reported in the Results section were response latency (time between presentation of the tone and response on the correct lever) and the percentage positive responses (number of responses made on the high reward lever divided by the total number of responses made and on the high reward and low reward levers). Each measure was averaged (by tone) across the whole session. Percentage omissions for each tone (trials where no lever press occurred during 20 s tone presentation divided by total completed trials) and number of premature responses across a session (trials where a response was made in the 5 s inter-trial interval before tone presentation) were also analysed. This data is not shown in the Results section as there were no significant differences between control and manipulations for premature responding or for omissions for the midpoint tone in any experiment (see S2 Table).

Cognitive bias index (CBI) was calculated for the midpoint tone only by subtracting the proportion of responses made on the low reward lever from the proportion of responses made on the high reward lever. This created a score between -1 and 1, where negative values represent a negative bias and positive values a positive bias. Change from baseline in CBI (control or vehicle probe test session minus the manipulation probe test session) was calculated to take into account individual differences in baseline CBI. Similarly, the change from baseline in distance to upper and lower boundaries is presented for diffusion model data. Change from baseline measures were analysed with one-sample *t*-tests with test values of zero (representing no change from baseline).

Diffusion model parameters analysed for each tone were distance to upper boundary (*a*_*+*_), distance to lower boundary (*a*_-_) and drift rate (*v*).

In Experiment 1, behavioural and model measures were analysed for all tones using two-way repeated measures analysis of variance (ANOVA) with tone and session as within-subjects factors. In Experiment 2, measures were analysed for the midpoint tone only. Diffusion model parameters were summarised across each part (pre-stress, stress and post-stress) by averaging values from individual sessions. Mixed ANOVAs were performed with one repeated measure (session) as the within-subjects factor and group as the between-subjects factor. A summary measure for CBI was analysed by calculating the area under the curve (AuC) over the six probe test sessions conducted during the stress and post-stress period. This and the diffusion model change from baseline measures were analysed with one-sample and independent samples *t*-tests. Independent samples *t*-tests were used to compare control independent component scores to projected scores for each negative affective state manipulation.

One-way repeated measures ANOVAs, paired *t*-tests or independent samples *t*-tests were performed as appropriate as post-hoc tests if significant effects were established. Huynh-Feldt corrections were used to adjust for violations of the sphericity assumption, and Levene’s test was used to correct for inequality of variances for ICA. Bonferroni correction was applied for multiple pairwise comparisons. For all statistical tests *α* = 0.05 was used, and all tests were conducted using SPSS 21.0.0.0 for Windows (IBM SPSS Statistics). Results are reported with the ANOVA *F-*value (degrees of freedom, error) and *p*-value as well as any post-hoc *p*-values. All graphs were made using Graphpad Prism 5.03 for Windows (Graphpad Software, USA).

## Results

### Behavioural results

#### Experiment 1: Acute induction of negative affective state

**Fig 2:**
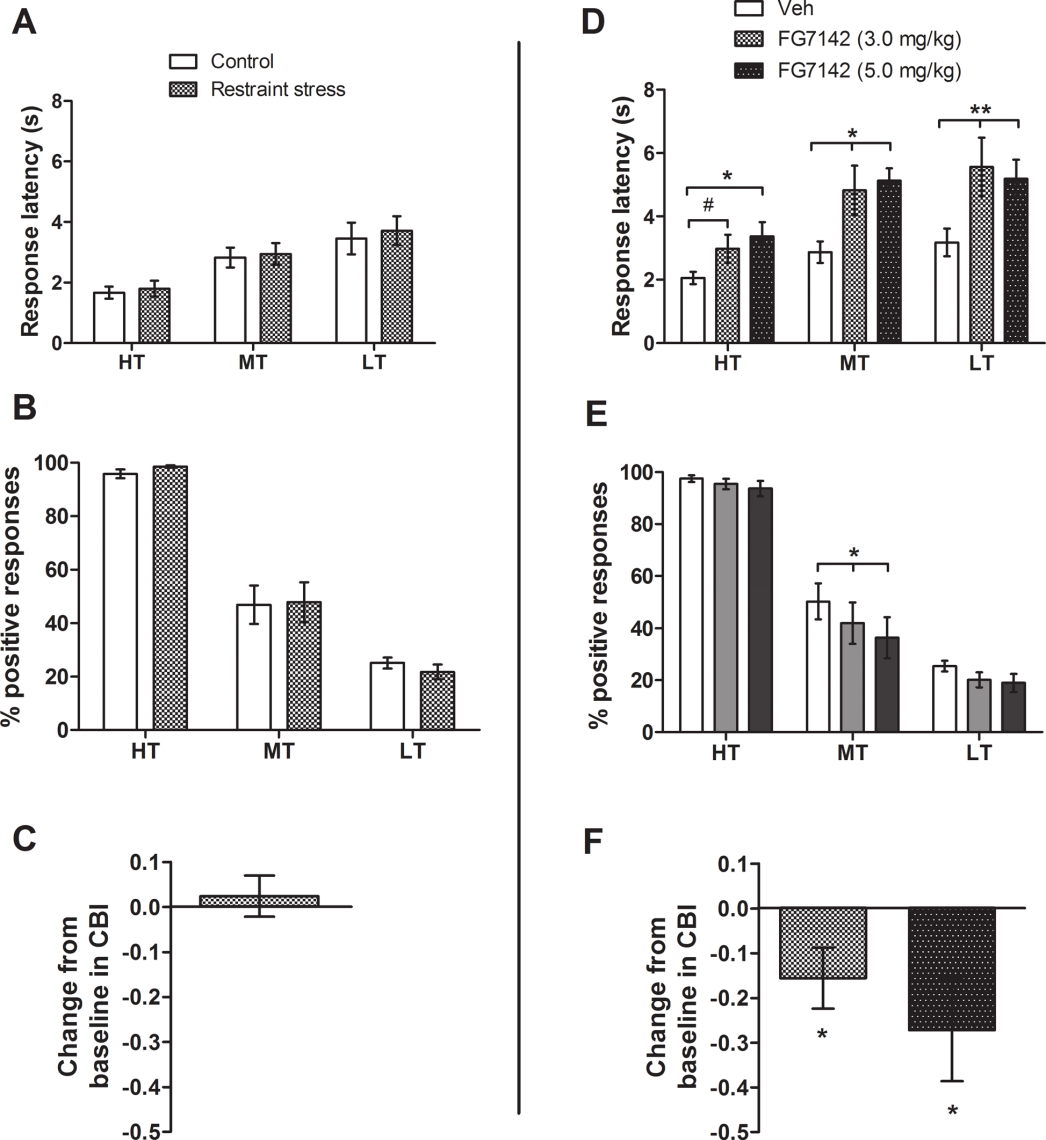
The effect of acute negative affective state manipulations on judgement bias. Acute restraint stress does not alter behaviour on the task, but an acute pharmacological negative affective state manipulation induces a negative bias. For the acute stress manipulation rats were placed in a restraint tube for 15 min immediately prior to testing on the task. For the acute pharmacological manipulation two doses of the anxiogenic drug, FG7142 (a GABA_A_ receptor partial inverse agonist), were administered acutely 30 min prior to testing on the task. (A) Acute restraint stress had no effect on the response latency, did not alter (B) the percentage positive responding, and there was no change in (C) change from baseline in cognitive bias index (CBI). (D) Both doses of FG7142 (3.0 and 5.0 mg/kg) increased response latency across all three tones. (E) FG7142 caused a significant reduction in percentage positive responding for the midpoint tone only for both doses. (F) This is supported by the negative change from baseline in CBI for both doses of FG7142. Data represent mean ± SEM; Acute restraint stress: *n* = 11; FG7142: *n* = 10. **p < 0.01, *p < 0.05, ^#^*p* < 0.06. Veh–vehicle; HT—high reward tone; MT—midpoint tone; LT—low reward tone.

For both manipulations, a main effect of tone was found for response latency (repeated measures ANOVAs: *F*s ≥ 8.63, *p*s ≤ 0.002), and the percentage positive responses (repeated measures ANOVAs: *F*s ≥ 74.67, *p*s < 0.001), indicating rats were able to discriminate between tones used. Rats were slower to respond to the midpoint and low reward tones compared to the high reward tone in each experiment (post-hoc pairwise comparisons: *p*s ≤ 0.033; Fig 2A and 2D). Rats made > 90% of responses on the high reward lever for the corresponding tone and approximately 25% positive responses for the low reward tone. On control/vehicle sessions, rats made approximately 50% responses on each lever during presentations of the midpoint ambiguous tone at baseline (Fig 2B and 2E).

For the acute restraint stress manipulation, eleven rats met criteria to be included in the analysis. There was no effect of restraint stress on any behavioural measure for any tone (Fig 2A–2C).

Ten rats were included in the analysis for the pharmacological negative affective state manipulation where the effect of an anxiogenic drug, FG7142 (a GABA_A_ receptor partial inverse agonist), on judgement bias was assessed. Results show a drug-induced negative judgement bias which increases in a dose-dependent manner. For response latency, a session*tone interaction (repeated measures ANOVA: *F*_4,36_ = 2.98, *p* = 0.032) and main effect of session (*F*_2,18_ = 9.02, *p* = 0.002) were observed. Post-hoc analyses indicated that 3.0 mg/kg FG7142 made rats slower to respond to the midpoint and low reward tones (post-hoc pairwise comparisons; midpoint: *p* = 0.012, low: *p* = 0.005) compared to controls, with a tendency towards the same effect at the high reward tone (post-hoc pairwise comparisons: *p* = 0.053; Fig 2D). The higher dose of FG7142 (5.0 mg/kg) made rats slower to respond to all tones (post-hoc pairwise comparisons; high: *p* = 0.016, midpoint: *p* = 0.001, low: *p* = 0.006; Fig 2D). A main effect of session was found for percentage positive responding (repeated measures ANOVA: *F*_1.41,12.72_ = 4.50, *p* = 0.044). Post-hoc tests showed for the midpoint ambiguous tone only, rats made fewer responses on the high reward lever following both doses of FG7142 compared to vehicle (3.0 mg/kg: *p* = 0.041, 5.0 mg/kg: *p* = 0.039; Fig 2E). For both doses, the change from baseline in CBI was also negative (one-sample *t*-tests; 3.0 mg/kg: *p* = 0.048, 5.0 mg/kg: *p* = 0.041; Fig 2F).

#### Experiment 2: Chronic induction of negative affective state

**Fig 3:**
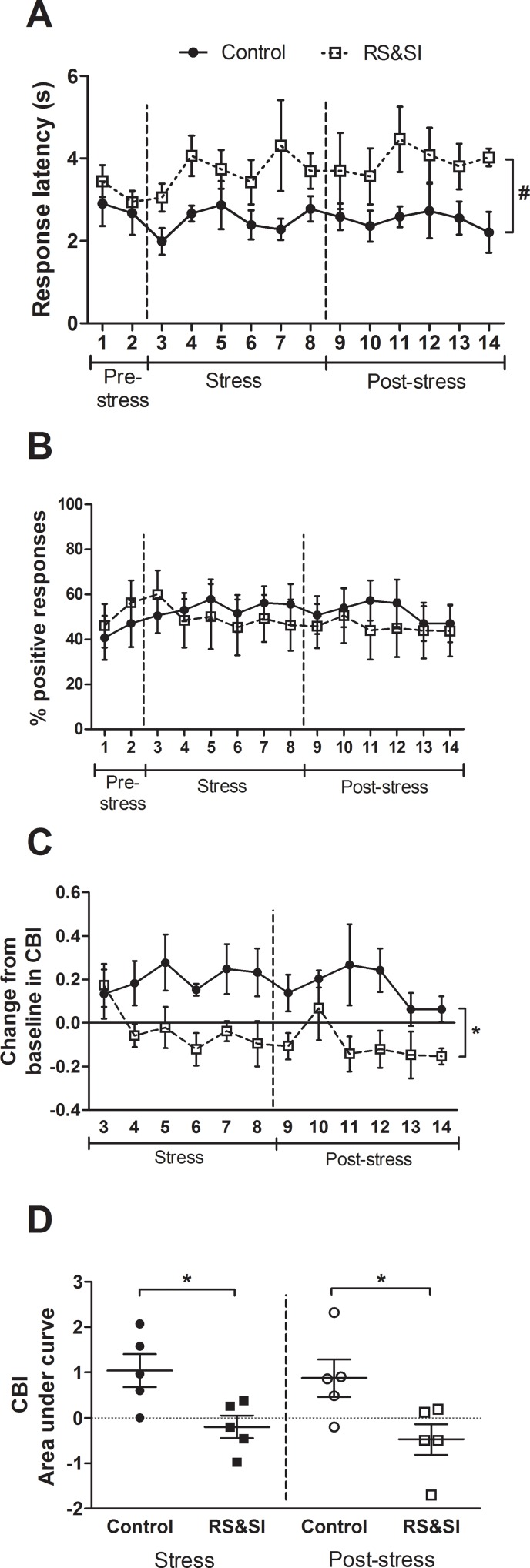
The effect of a chronic negative affective state manipulation on judgement bias. Rats assigned to the stress group experienced repeated restraint stress and social isolation (RS&SI) for three weeks. Twice weekly test sessions were conducted one week prior to stress (pre-stress; sessions 1–2), for the three weeks during RS&SI (stress; sessions 3–8) and for three weeks following return to control conditions (post-stress; sessions 9–14). Data are shown for the midpoint tone only. (A) RS&SI seemed to make rats slower to respond during stress and post-stress sessions. (B) There was no clear effect of RS&SI on the percentage positive responding. (C) Rats in the RS&SI group showed a significantly negative change from baseline in cognitive bias index (CBI) compared to controls. (D) Summarising the change from baseline in CBI over the six stress and six post-stress session by calculating the area under the curve for this measure for individual rats indicated that RS&SI caused a significant negative bias, during both the stress and post-stress periods. Data represent mean ± SEM; *n* = 5 per group. *p < 0.05, ^#^*p* < 0.06.

Five rats per group were included in the analysis for this experiment. The results show that compared to controls, chronic stress causes more negative judgement bias in this task, which does not reverse after three weeks of control conditions. There were no differences between the control and RS&SI group for any variables analysed in the two pre-stress probe test sessions (see sessions 1–2 on Fig 3A and 3B and S2 Table). Although not significant, RS&SI seemed to increase response latency for the midpoint tone (pairwise group difference: *p* = 0.055; Fig 3A). There was a trend towards a session*group interaction for percentage positive responding at the midpoint tone (repeated measures ANOVA: *F*_13,104_ = 1.68, *p* = 0.077; Fig 3B), but no significant interaction for change from baseline in CBI. However, there was a pairwise group difference (*p* = 0.023) for this measure, which indicates that CBI in the RS&SI group became more negative. This can be seen during both stress and post-stress periods (Fig 3C). Despite a lack of significance, visual inspection of the change in CBI on individual sessions suggests that this bias does not occur immediately following the onset of chronic stress, instead occurring one week later (see session 3 compared to session 4 on Fig 3C). There was a smaller AuC for change from baseline in CBI for the RS&SI group compared to the control group during the stress period (independent samples *t*-test: *p* = 0.022; Fig 3D), indicating reduced CBI scores. This overall reduction in CBI was not reversed by return to control conditions, as the AuC for change from baseline in CBI during the three weeks post-stress was still reduced in RS&SI rats compared to controls (independent samples *t*-test: *p* = 0.035; Fig 3D).

### Modelling results

#### Model fit validation

**Fig 4:**
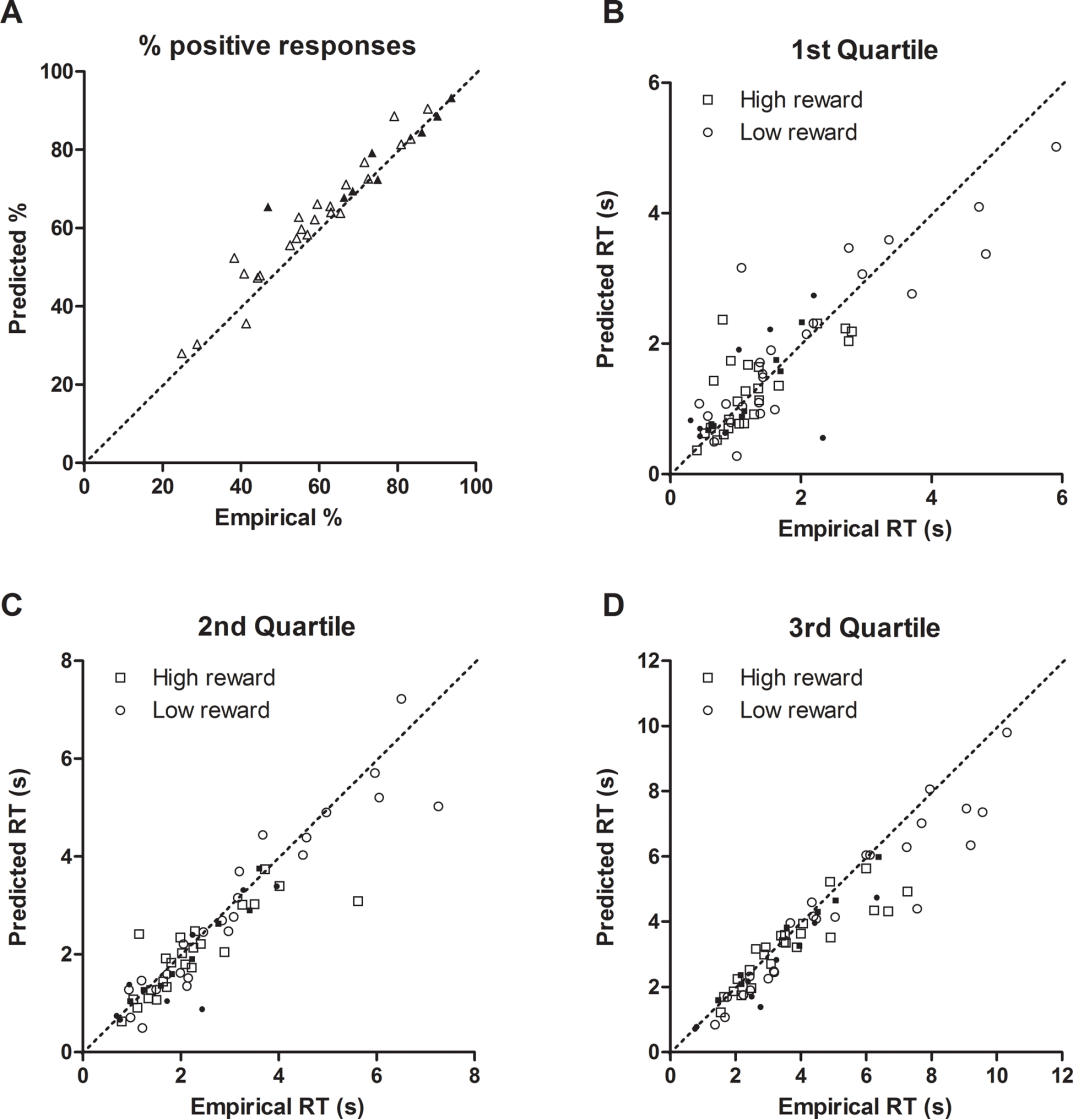
Diffusion model fit validation comparing empirical and predicted data. Graphical analysis of model fit comparing behavioural (empirical) and model (predicted) data. Data points lie close to the main diagonal (dotted line) for both (A) the percentage of positive responses and (B/C/D) the values for response times (RTs) from the three quartiles of the cumulative distribution functions for the midpoint tone, indicating good model fit. For RTs, squares represent the distribution for high reward responses, while circles represent the distribution for low reward responses. Each symbol corresponds to the data value for one rat on one probe test session. Filled symbols represent rats that were then consistently excluded from all behavioural experiments, and were therefore not included in any further analysis. Importantly, these points are randomly distributed amongst the data.

Model fit was assessed using behavioural data from two probe test sessions (shown in S1 Fig). The parameter combination that produced best model fit based on both fast-dm outputted goodness of fit *p*-values and graphical inspection was selected for modelling of experimental behavioural data. The goodness of model fit *p*-values for the selected model for each rat were *p* > 0.05 (mean ± SEM = 0.891 ± 0.015). The predicted RT distribution from diffusion model output from the control data point with the median goodness of fit *p*-value (*p* = 0.862) along with corresponding behavioural RT data are shown in Fig 1. Graphical inspection was conducted for all three tones as some parameters are fit separately to each tone, but data is only shown for the midpoint ambiguous tone. Graphical inspection of the percentage of positive responses for behavioural data (empirical) compared with the model (predicted) are shown in Fig 4A. The comparison of RTs corresponding to the first, second and third quartiles of empirical and predicted CDFs are shown in Fig 4B–4D. Data points lie close to the main diagonal in all four plots, indicating good model fit. Model fit validation was conducted for all rats (*n* = 16), however some rats were consistently excluded from all behavioural manipulation experiments for failing to meet criteria. Data points for these rats are indicated by filled in points on the graph. There were no differences between sessions or groups for the percentage of excluded rapid responses for any experimental manipulations (S3 Table).

#### Modelling of experimental data

The diffusion model was fit for each individual rat to behavioural data from each probe test session. Three parameters—distance to the upper boundary (*a*_*+*_), distance to the lower boundary (*a*_*-*_) and drift rate (*v*)—were compared between control/vehicle and negative affective state manipulations in both experiments. There were no differences in other model parameters (*t*_*0*_, *d* and *szr*) between control and negative state manipulation data for any experiment (S4 Table).

#### Experiment 1

**Fig 5:**
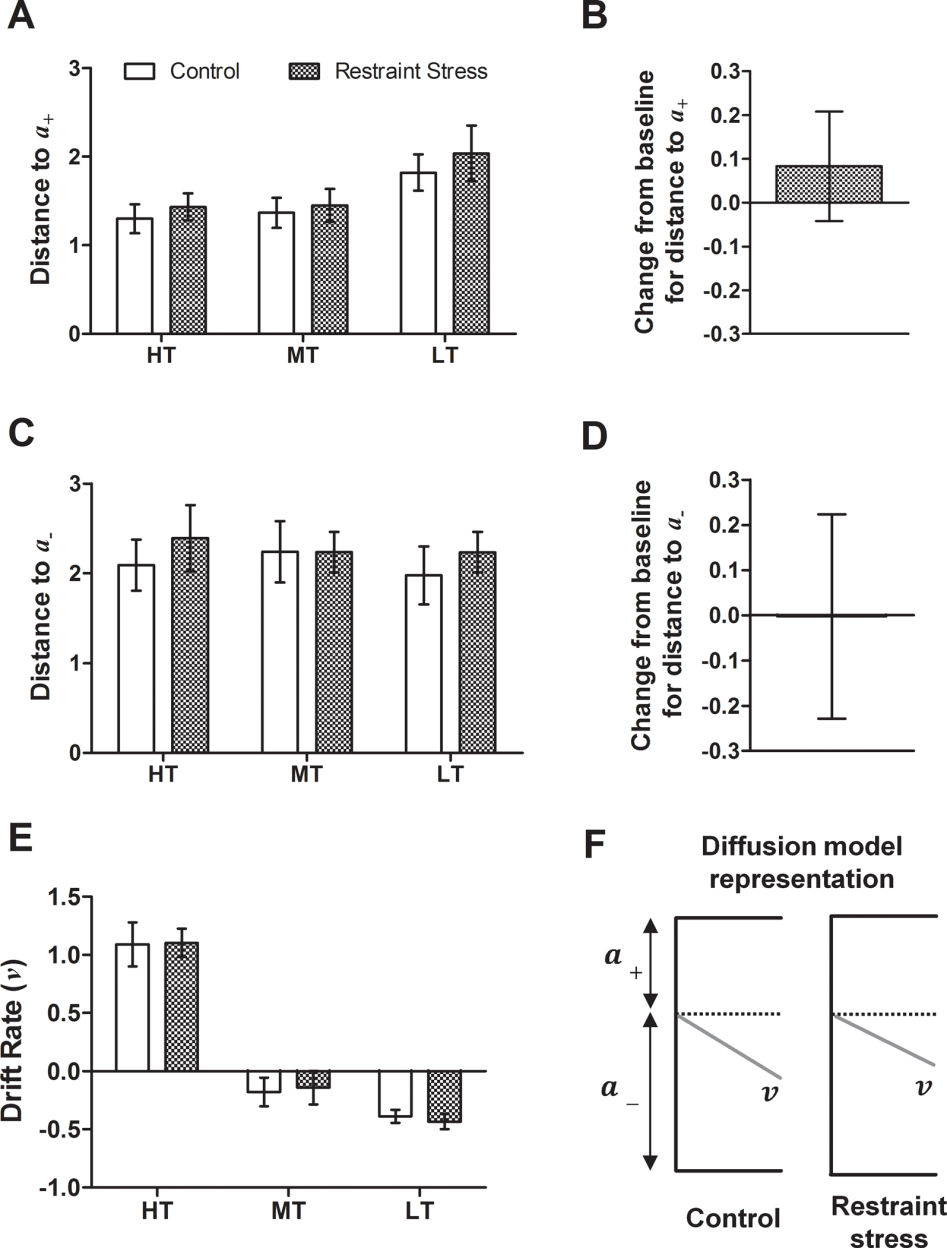
Diffusion model parameters for data from the acute restraint stress manipulation. Three parameters—distance to the upper boundary (*a*_*+*_; A/B), distance to the lower boundary (*a*_-_; C/D) and drift rate (*v*) were analysed for each tone. Change from baseline (control session) for *a*_*+*_ and *a*_*-*_ was also calculated for the midpoint tone only to take into account individual differences in underlying bias. Acute restraint stress did not alter (A/B) *a*_*+*_; (C/D) *a*_*-*_ or (E) *v*. (F) Diagrammatic representation of diffusion model parameters for the midpoint tone only for this manipulation. Illustrations are to scale representing mean values for each measure, and are aligned to decision starting point (dotted line). Drift rate is scaled 5:1. Data represent mean ± SEM; *n* = 11. HT—high reward tone; MT—midpoint tone; LT—low reward tone.

**Fig 6:**
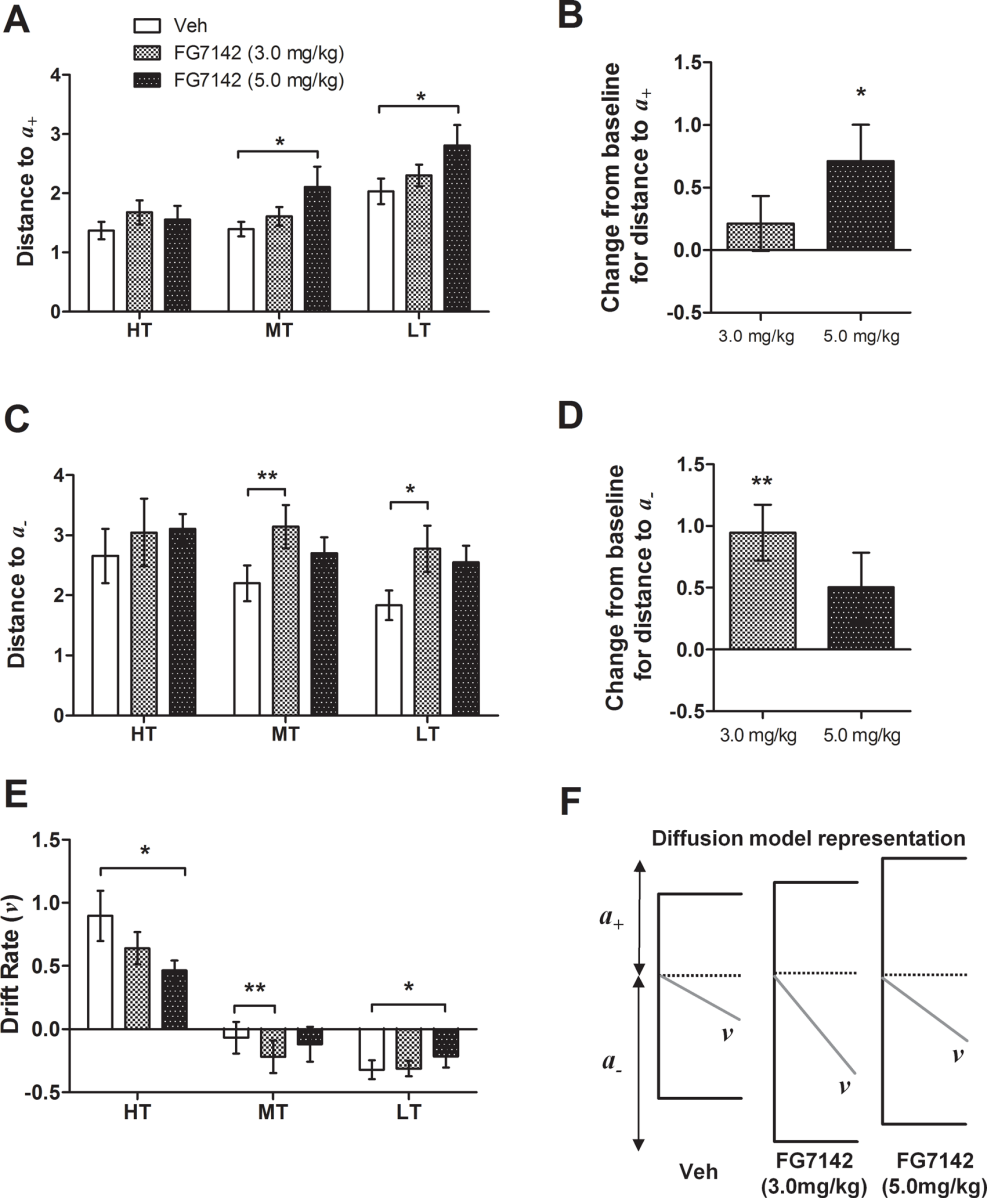
Diffusion model parameters for data from acute FG7142 treatment. Three parameters—distance to the upper boundary (*a*_*+*_; A/B), distance to the lower boundary (*a*_-_; C/D) and drift rate (*v*) were analysed for each tone. Change from baseline (control session) for *a*_*+*_ and *a*_*-*_ was also calculated for the midpoint tone only to take into account individual differences in underlying bias. (A/B) The higher dose of FG7142 (5.0 mg/kg) only caused a significant increase and positive change from baseline in *a*_*+*_ for the midpoint and low reward tones. (C/D) The lower dose of FG7142 (3.0 mg/kg) only caused a significant increase and positive change from baseline in *a*_*-*_ for the midpoint and low reward tones. (E) FG7142 differentially altered *v* dependent on both tone and dose. (F) Diagrammatic representation of the diffusion model parameters for the midpoint tone only for this manipulation. Illustrations are to scale representing mean values for each measure, and are aligned to decision starting point (dotted line). Drift rate is scaled 10:1 to more clearly illustrate changes. Graph data represent means ± SEM; *n* = 10. **p < 0.01, *p < 0.05. Veh–vehicle; HT—high reward tone; MT—midpoint tone; LT—low reward tone.

For both manipulations, there were main effects of tone for *v* (repeated measures ANOVAs: *F*s ≥ 23.46, *p*s < 0.001), which confirms rats could discriminate between tones used. The midpoint tone had the drift rate closest to zero, which is consistent with this tone failing to provide any information that aids discrimination (see control/vehicle data on Figs 5E and 6E). There was a main effect of tone for *a*_*+*_ for both acute manipulations (repeated measures ANOVA: *F*s ≥ 7.98, *p*s ≤ 0.009; see control/vehicle data on Figs 5A and 6A). This indicates *a*_*+*_ was smallest for the high reward tone, and larger for both midpoint and low reward tones. The opposite pattern was seen for *a*_*-*_ for the pharmacological manipulation (repeated measures ANOVA: *F*_2,18_ = 4.34, *p* = 0.029), but not for the stress manipulation.

Consistent with the behavioural data, acute restraint stress did not alter any diffusion model parameters for any tone. This was apparent both from overall analyses (Fig 5A, 5C and 5E) and from the comparison of *a*_*+*_ and *a*_*-*_ to baseline values for the midpoint tone (Fig 5B and 5D). This confirms that acute restraint stress did not modify decision making processes. Fig 5F shows a diagrammatic representation of diffusion model parameters for the midpoint ambiguous tone during control and acute restraint stress sessions.

FG7142 had a dose-dependent effect on model parameters. *a*_*+*_ was increased for the higher dose of FG7142 (5.0 mg/kg) only, for both the midpoint and low reward tones (repeated measures ANOVA main effect of session: *F*_2,18_ = 5.21, *p* = 0.016, and significant post-hoc pairwise comparisons; midpoint: *p* = 0.038, low: *p* = 0.034; Fig 6A). This is also illustrated in Fig 6B, where compared to baseline, *a*_*+*_ is larger for 5.0 mg/kg of FG7142 (one-sample *t*-test: *p* = 0.038). This indicates more information was required to cross the threshold corresponding to a choice to press the high reward lever, indicative of a negative judgement bias. There was an increase in *a*_*-*_ for the lower dose of FG7142 (3.0 mg/kg) only, again for both the midpoint and low reward tones (repeated measures ANOVA main effect of session: *F*_2,18_ = 4.31, *p* = 0.030, and significant post-hoc tests; midpoint: *p* = 0.002, low: *p* = 0.008; Fig 6C). This is confirmed in Fig 6D, which shows *a*_*-*_ is greater compared to baseline for 3.0 mg/kg of FG7142 (one-sample *t*-test: *p* = 0.002). FG7142 had dose- and tone-dependent effects on *v* (repeated measures ANOVA session*tone interaction: *F*_2.27, 20.45_ = 4.43, *p* = 0.022). The higher dose of FG7142 (5.0 mg/kg) caused changes to *v* for the high reward and low reward tones (post-hoc tests; high: *p* = 0.040, low: *p* = 0.046; Fig 6E), whereby the magnitude was decreased (*v* was closer to zero). The lower dose of FG7142 (3.0 mg/kg) had a specific effect on *v* at the midpoint tone, causing *v* to become more negative (post-hoc pairwise comparison: *p* = 0.001; Fig 6E). Fig 6F shows a diagrammatic representation of the effect of both doses of FG7142 on diffusion model parameters for the midpoint ambiguous tone.

#### Experiment 2

**Fig 7:**
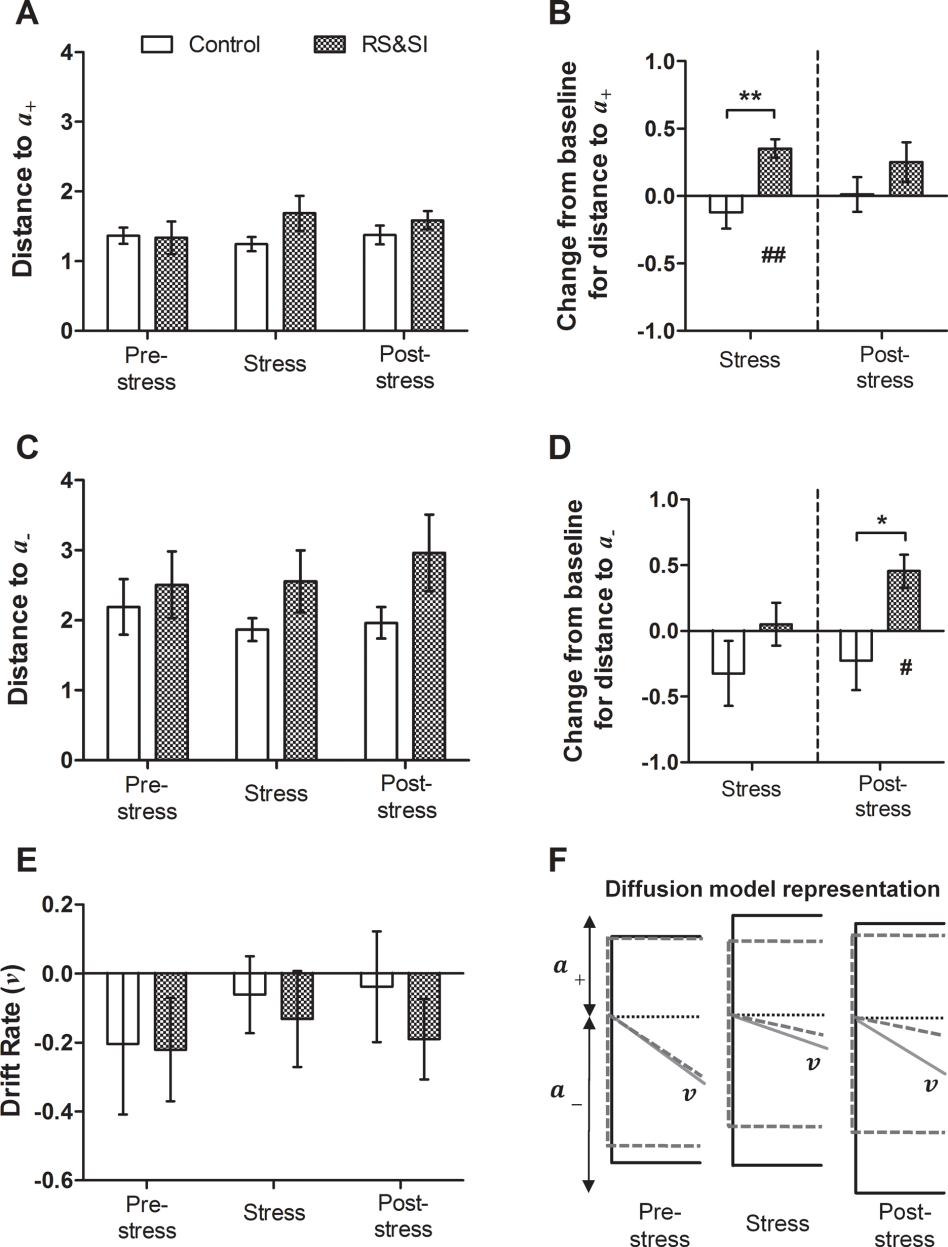
Diffusion model parameters for data from Experiment 2. Three parameters—distance to the upper boundary (*a*_*+*_; A/B), distance to the lower boundary (*a*_-_; C/D) and drift rate (*v*) were analysed for each tone. Change from baseline (control session) for *a*_*+*_ and *a*_*-*_ was also calculated for the midpoint tone only to take into account individual differences in underlying bias. Data shown are averages for all probe sessions during that experimental period. (A) Changes in *a*_*+*_ in the restraint stress and social isolation (RS&SI) group compared to control group were unclear from the overall analysis of this measure. (B) However, during stress only, change from baseline in *a*_*+*_ was positive in the RS&SI group, and significantly larger compared to controls. (C) The effect of RS&SI on the *a*_*-*_ was again unclear from the overall analysis. (D) During the post-stress period only, a positive change from baseline in *a*_*-*_ was seen in the RS&SI group, and was also significantly larger compared to controls. (E) There was no difference in *v* between the RS&SI and control group during any stage of the manipulation. (F) Diagrammatic representation of diffusion model parameters for the control (dashed lines) and RS&SI group (solid lines) for the midpoint tone only for this manipulation. Illustrations are to scale representing mean values for each parameter, and are aligned to decision starting point (dotted line). Drift rate is scaled 5:1. Graph data represent mean ± SEM; *n* = 5 per group. Independent samples *t*-tests to compare groups: **p < 0.01, *p < 0.05; one-sample *t*-tests to compare to baseline: ^##^*p* < 0.01, ^#^*p* < 0.05.

RS&SI had a differential effect on model parameters during the stress and post-stress periods. There was a session*group interaction (mixed ANOVA: *F*_2,16_ = 3.77, *p* = 0.046; Fig 7A) for *a*_*+*_, however post-hoc tests did not reveal any significant differences on individual session types. Calculating the change from baseline in *a*_*+*_ revealed an increase in this measure in the RS&SI group during stress only (Fig 7B). This difference was compared to their own baseline (one-sample *t*-test: *p* = 0.006), and compared to controls (independent samples *t*-test: *p* = 0.009). There was also a session*group interaction (mixed ANOVA: *F*_2,16_ = 3.92, *p* = 0.041) for *a*_*-*_. Again, there were no significant differences between the control and RS&SI groups on individual session types (Fig 7C). The change from baseline in this measure clarifies the effect of RS&SI, with an increase being seen in *a*_*-*_ during the post-stress period only, compared to baseline (one-sample *t*-test: *p* = 0.022) and compared to controls (independent samples *t*-test: *p* = 0.030; Fig 7D). There was no effect of RS&SI on *v* during any of the three experimental periods (Fig 7E). A diagrammatic representation of the changes to diffusion model parameters caused by RS&SI is shown by the solid lines in Fig 7F.

### Independent Component Analysis

**Fig 8:**
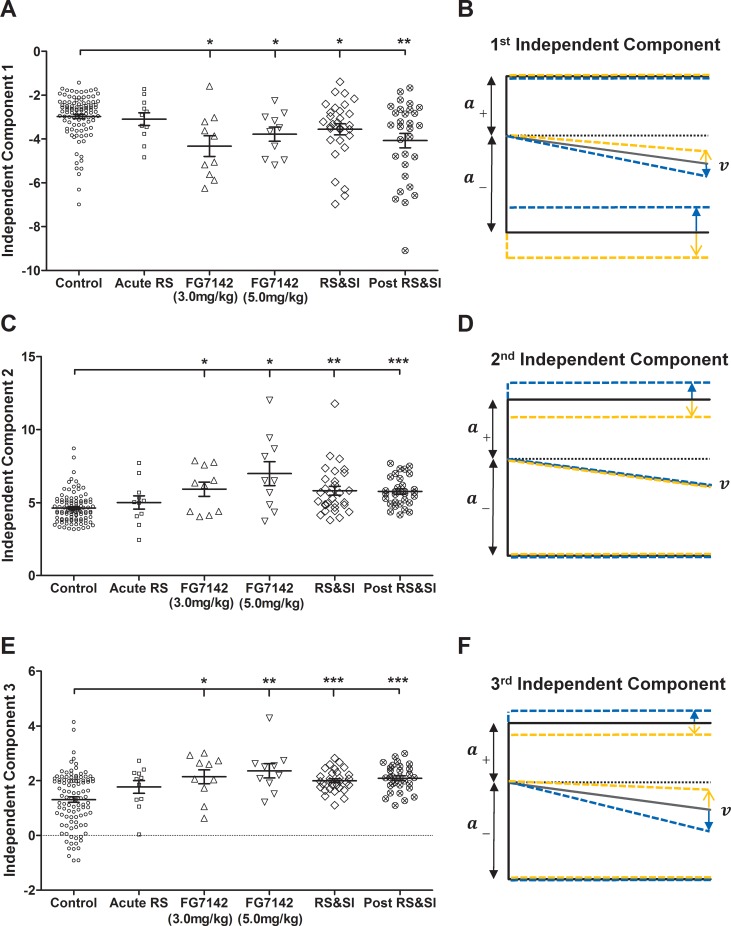
Independent Component Analysis scores calculated from diffusion model parameter data. Independent Component Analysis (ICA) was conducted on distance to the upper boundary (*a*_*+*_), distance to the lower boundary (*a*_-_) and drift rate (*v*) data for control data for the midpoint tone only. Negative manipulation data was then projected using the independent components found. (A/C/E) Independent component scores showing the distribution of parameter data for each manipulation. Scores for all three independent components are significantly different for both doses of FG7142 and the stress and post-stress periods of Experiment 2. Control data from all negative affective state manipulations are shown together. Each symbol represents the data from a single session for a single rat for that manipulation, mean ± SEM is shown by the black line and error bar for each manipulation. (B/D/F) Diagrammatic representations of the direction of change of diffusion model parameter values for control data (yellow) and negative affective state manipulation data (blue) described by the independent component. Black lines represent mean values for parameters from all data (control and negative manipulations). (B) The first independent component describes increased expectation for the low reward and more negative interpretation of the ambiguous cue for negative affective state manipulations compared to control. (D) The second independent component reflects reduced anticipation for the high reward for negative manipulation data. (F) The third independent component comprises reduced expectation for the high reward and more negative interpretation of the ambiguous cue for the negative affective state manipulations. ***p < 0.001, **p < 0.01, *p < 0.05. RS—restraint stress; RS&SI—restraint stress and social isolation.

ICA of diffusion model control data for *a*_*+*_, *a*_*-*_ and normalised *v* revealed three distinct components can explain decision making behaviour. Scores for negative affective state manipulations (that were behaviourally significant) that were projected into the independent component space were significantly different from control scores for all three components (Fig 8A, 8C and 8E). Scores for the first independent component were more negative compared to controls for both doses of FG7142 and for stress and post-stress periods during RS&SI (independent samples *t*-tests: *p*s ≤ 0.012). Using values from the separating matrix (***W***) for the first component (*a*_*+*_ = -0.018; *a*_*-*_ = -0.916; *v* = -0.400) it can be determined that this reflects reduced distance to the low reward boundary and more negative drift rate for negative affective state manipulations compared to control data. This is shown in Fig 8B by a diagrammatic representation of the directions of change in diffusion model parameters explained by the component (the same diagrams are shown for the second and third components in Fig 8D and 8F). Scores for the second (Fig 8C) and third independent components (Fig 8E) were more positive for negative affective state manipulations (both doses of FG7142 and for the RS&SI group during stress and post-stress) than for control data (independent samples *t*-tests: *p*s ≤ 0.018 for component 2 and *p*s ≤ 0.012 for component 3). Values from ***W*** for the second component (*a*_+_ = 0.979; *a*_*-*_ = 0.205; *v* = 0.021) indicate that there is an increased distance to the high reward boundary for negative affective state manipulation data (Fig 8D). For the third component, values from ***W*** (*a*_*+*_ = 0.463; *a*_*-*_ = 0.060; *v* = -0.885) show that negative affective state data is explained by a combination of increased distance to the high reward boundary and a more negative drift rate (Fig 8F).

## Discussion

This study has combined a high versus low reward ambiguous-cue interpretation task with computational modelling to investigate the decision making processes underlying negative judgement bias. Negative judgement bias was induced by the anxiogenic drug FG7142 and chronic RS&SI, but acute restraint stress failed to cause a measurable negative affective state change. Diffusion model analysis revealed that negative judgement bias was mainly due to increases in distance to boundaries and changes in distances relative to decision starting point. ICA of the diffusion model parameters revealed the negative bias could be explained by three distinct components that are related to increased expectation of the low reward, reduced anticipation of the high reward and more negative interpretation of the ambiguous cue.

### Behavioural effects of negative affective states

Restraint stress has been widely used as a rodent model of psychological stress (reviewed by [45]) and is therefore expected to cause temporary negative affect. The absence of behavioural changes following acute restraint stress (Fig 2A–2C) may be because temporary negative affect is quickly alleviated once rats are in the positive context of the R-R task, or that a single brief period of restraint stress (15 min) was not a potent enough stressor to alter behaviour. Some support for this is provided through visual inspection of the chronic RS&SI manipulation data, where a negative shift in CBI does not occur immediately following the onset of stress (see session 3 compared to 4 in the RS&SI group on Fig 3C). This suggests acute restraint stress has insufficient negative impact to cause behavioural changes during the task, but has an additive effect over time that induces negative affective state.

Acute pharmacological induction of negative affective state on judgement bias has not specifically been tested, although acute treatments with noradrenergic drugs have induced negative biases [21, 22, 46], similar to the effect seen with FG7142 in Experiment 1 (Fig 2D–2F). FG7142 treatment increased response latency to reference tones as well as the ambiguous tone suggesting a general decrease in motivation that may have contributed to the effect on bias. However, no differences were seen in percentages of high and low reward responses to either reference tone (Fig 2E), suggesting the observed bias is caused by the change in affective state.

Induction of a negative affective state through RS&SI in Experiment 2 (Fig 3) replicates findings on R-P tasks where chronic mild stress [20], chronic restraint stress [25], and chronic psychosocial stress [24] caused negative judgement biases. Interestingly, the negative bias induced by RS&SI was not reversed in the three weeks post-stress. Rygula et al. [25] found that the more negative CBI seen during chronic restraint stress was reversed one week post-stress. Other studies have not reported judgement bias following stress. Visual inspection of Fig 3C indicates that one week post-stress (session 10) CBI did shift back towards baseline, but this was not maintained in later sessions. This could suggest that measuring judgement bias over longer time periods is required to identify persistent effects due to chronic stress, or alternatively could indicate that for this type of stress manipulation, return to control conditions (pair housing) may itself be a stressor. Previous studies have shown that chronic stress experienced during adolescence [47] or the juvenile period [48] causes long-term changes in judgement bias measured in adult rats. This supports the conclusion that chronic RS&SI causes enduring judgement bias changes, but further experimentation would be required to confirm this.

The advantages of using reward-based tasks rather than R-P tasks span ethical and practical considerations. The R-R task avoids the use of aversive training methods, and reduces potential confounds linked to animals being exposed to repeated aversive (albeit escapable) foot shock [49]. This helps to minimise suffering caused to laboratory animals during experiments, which is favourable for animal welfare [14]. Practically, reward-based tasks mean that rats can be trained more quickly (22 days for most rats in this task compared to an average of > 40 days for R-P tasks; calculated from Parker et al. [50]), as rats will learn to press a lever to obtain food reward within a few days, however experience much more difficulty in acquiring an active avoidance response [50, 51]. Use of a reward-based task may also restrict the circuits involved in decision making to those regulating reward processing. Computational models are inherently simplified reproductions of actual neural processes, and so simplifying the neural systems involved increases the likelihood that a model will effectively recapitulate the behaviour of the system.

### Modelling negative judgement biases using the diffusion model

Application of the diffusion model to behavioural data enabled exploration of the decision making processes altered during negative affective state. Acute treatment with FG7142 caused dose-dependent changes in distances to boundaries and drift rate. The higher dose (5.0 mg/kg) reduced drift rates for both reference tones (Fig 6E), suggesting rats found the task more difficult and were not able to accumulate information as rapidly. Both doses caused increases in boundary separation (Fig 6F), which corresponds to more conservative decision making. This was also seen both during and after RS&SI in Experiment 2 (Fig 7F). Stress and anxiety are intricately linked, with clinical studies associating stressful life experiences with increased likelihood of anxiety (e.g. [52, 53]). In rodents chronic stress induces anxiety-like behaviour [54–57], and FG7142 is an anxiogenic drug [58, 59]. Therefore, increased boundary separation could be a consequence of increased anxiety. Diffusion modelling has not been used with behavioural data from clinically anxious patients, although in a lexical decision task the boundary separation parameter was increased (although not significantly) in dysphoric compared to non-dysphoric participants [29]. This matches the direction of change found in this study (Figs 6F and 7F).

In Experiment 2, diffusion modelling revealed that different processes underlie negative judgement bias measured during RS&SI–increased distance to the high reward boundary–compared with post-stress: increased distances to boundaries overall combined with a slightly but not significantly more negative drift rate (Fig 7B, 7D and 7F). Stress causes long-term changes in the brain, including altering neurogenesis and neuronal morphology (reviewed by [60]). This could provide a potential mechanism for these differences, although additional experiments would be required to investigate further.

The diffusion model has not previously been applied to data from rodent behavioural tasks, having been used extensively with human two-choice RT tasks (reviewed in [61]), and more recently with non-human primate behavioural tasks [62–64]. Animal behaviour can be highly variable, meaning subtle changes can be difficult to detect (see [65] for an example). Diffusion modelling can help to overcome this, as incorporation of entire RT distributions for correct and error responses, and modelling individual animals separately permits more thorough interpretation of data. Model fit *p*-values (mean ± SEM = 0.819 ± 0.015) and graphical model fit validation (Fig 4) demonstrate that with the parameter conditions used, data from this rodent behavioural task can be effectively modelled with the diffusion model.

Diffusion modelling also allows further conclusions to be drawn than could be from analysis of behavioural data alone. Despite the same negative behavioural judgement bias being observed following both acute (FG7142) and chronic (RS&SI) induction of negative affective state, diffusion modelling suggests that this negative bias is brought about by alterations to different underlying decision making process. For the acute manipulation, negative bias was explained by changes in both distances to boundaries and in drift rate, whereas for the chronic manipulation no significant changes in drift rate occurred. Different combinations of parameter changes also explain the negative bias seen during stress compared to post-stress in Experiment 2.

ICA of diffusion model parameters clarified the main hidden components that differ in decision making processes related to negative judgement bias. All three independent components were significantly different between control and negative affective state manipulation data, indicating that all three components contribute to negative judgement bias. The first component corresponds to a more negative drift rate and a smaller distance between the decision starting point and the low reward boundary for negative affective state manipulations (Fig 8A and 8B). This can be interpreted as more negative interpretation of the ambiguous cue and an increased expectation of the low reward, meaning decisions about the ambiguous cue are much more likely to reach the low reward boundary. The second component reveals an increased distance to the high reward boundary during negative affective state manipulations (Fig 8C and 8D), which corresponds to reduced expectation of the high reward. The third component comprises increased distance from the decision starting point to the high reward boundary and a more negative drift rate for the negative affective state manipulations compared to control (Fig 8E and 8F). As explained above, these parameter changes reflect reduced anticipation for the high reward and more negative interpretation of the ambiguous cue.

Altogether, ICA suggests that negative judgement bias can be explained overall by more pessimistic decision making, which matches human behavioural data that has shown people with anxiety and depression exhibit more pessimistic interpretation of ambiguous information [66, 67]. However, the particular combination of parameters changes found suggest that there are distinct processes involved within this. Specifically, there is a dissociation between reduced anticipation of more positive outcomes (high reward) and increased anticipation of more negative outcomes (low reward) combined with more negative interpretation of the ambiguous cue. In humans, antidepressants cause specific increases in positive responding on tasks measuring affective bias [68]. This same distinction has also been seen in other rodent ambiguous-cue interpretation tasks, where negative bias has specifically been related to decreases in positive responding to ambiguous cues [20, 21]. Identification of specific changes in perception of more positive versus more negative outcomes could only be distinguished in these tasks through observing responses to multiple ambiguous cues, which decreases the number of times individual cues can be presented, hence reducing statistical power. The use of ICA allows this dissociation to be investigated in judgement bias tasks that utilise a single (midpoint) ambiguous cue, enabling the task to have both higher statistical power and increased sensitivity [69]. ICA provides support for the idea put forward by Enkel et al. [21] that reduced positive and increased negative responding may be separate phenomena that independently contribute to bias. Although the manipulations conducted in this study are complex with the induced negative bias comprising of both of these independent phenomena, the use of ICA with diffusion modelling demonstrated here provides a framework for use in future behavioural studies. This approach could be applied to alternative pharmacological and neural manipulations that alter affective state to identify the relative contributions of these two separate phenomena in observed behaviour, and therefore enable identification of specific neurotransmitters, receptors or brain areas that are important for these processes. This also links to the many studies showing that there are different patterns of alterations of processing of positive versus negative stimuli and information in patients with anxiety compared to those with depression, and that this may be due dysfunction in different neural circuits (see [4, 5, 70] for detailed reviews).

These experiments provide new insight into the decision making processes that underlie judgement bias following negative affective state manipulations in rats. Increased anticipation of more negative and reduced anticipation of more positive outcomes alongside more pessimistic interpretation of an ambiguous cue underlie behavioural negative affective bias induced by FG7142 and RS&SI. The novel application of the diffusion model and ICA to rodent behavioural data enabled specific changes to components of decision making to be elucidated and provides a potentially valuable method for investigating the different neural pathways involved in affective bias. Patients with mood disorders show abnormal processing of rewarding [7, 71] and aversive [72] stimuli. Studying the different manifestations of affective biases in response to changes in affective state in tasks with both positively and negatively valenced outcomes will extend this work and enable greater understanding of the aberrant decision making processes which contribute to anxiety and depression [73–76].

## Supporting Information

S1 Fig Behavioural data used for diffusion model fitting. Two probe tests were conducted prior to experimental manipulations to use for diffusion model fitting and validation. (A) Latency to respond to each tone. (B) The percentage of positive responses made for each tone. Data represent mean ± SEM; *n* = 16. HT—high reward tone; MT—midpoint tone; LT—low reward tone.
(TIF)

S1 File Supporting Materials and Methods. (DOCX)

S1 Table Training stages and advancement criteria. Summary of the stages used to train rats on the reward-reward operant ambiguous-cue interpretation task. The criteria column specifies the criteria rats were required to meet before they could advance to the next stage of training. Training time shows the maximum length of time necessary to meet criteria for each stage. Training time is given in both sessions and days, as some training stages were conducted twice per day. ITI–inter-trial interval. Between the end of training and the start of experimental manipulations another eight weeks elapsed which included the following: testing to provide data for diffusion model fitting and validation and to ensure stable responding to ambiguous tones during repeated probe tests over time (four weeks in total; data used for diffusion model fitting is shown in S1 Fig); a break with no testing during which rats received *ad libitum* food in the home cage (two weeks); and re-baseline sessions following the break (two weeks).
(DOCX)

S2 Table Amount of premature responses and percentages of omissions for Experiments 1 and 2. Data for number of premature responses made across the whole session (trials where a response was made in the 5 s ITI before tone presentation) and percentage of omissions for each tone (trials where no lever press occurred during 20 s tone presentation divided by total completed trials). These are data are separated into experiment and manipulation/group. * denotes significant difference (*p*<0.05) compared to control (repeated measures ANOVA and post-hoc test). All values are mean ± SEM.
(DOCX)

S3 Table Percentages of rapid responses removed before diffusion modelling. The percentage of rapid responses that were removed from behavioural data before diffusion model analysis. Rapid responses were defined as responses occurring with a latency of less than 200 ms. All values are mean ± SEM.
(DOCX)

S4 Table Other diffusion model parameters for Experiments 1 and 2. Data for other parameters fit by the diffusion model across all tones: the across-trial variability in decision starting point (*szr*) the non-decision time (*t*_*0*_) and the difference between the two responses in speed of execution (*d*). These data are separated into experiment and manipulation/group. All values are mean ± SEM.
(DOCX)
