# Supplementary figures and images for: Diffusion Modelling Reveals the Decision Making Processes Underlying Negative Judgement Bias in Rats
Source: PLoS One. 2016 Mar 29;11(3):e0152592. doi: 10.1371/journal.pone.0152592 (PMC4811525; doi:10.1371/journal.pone.0152592)

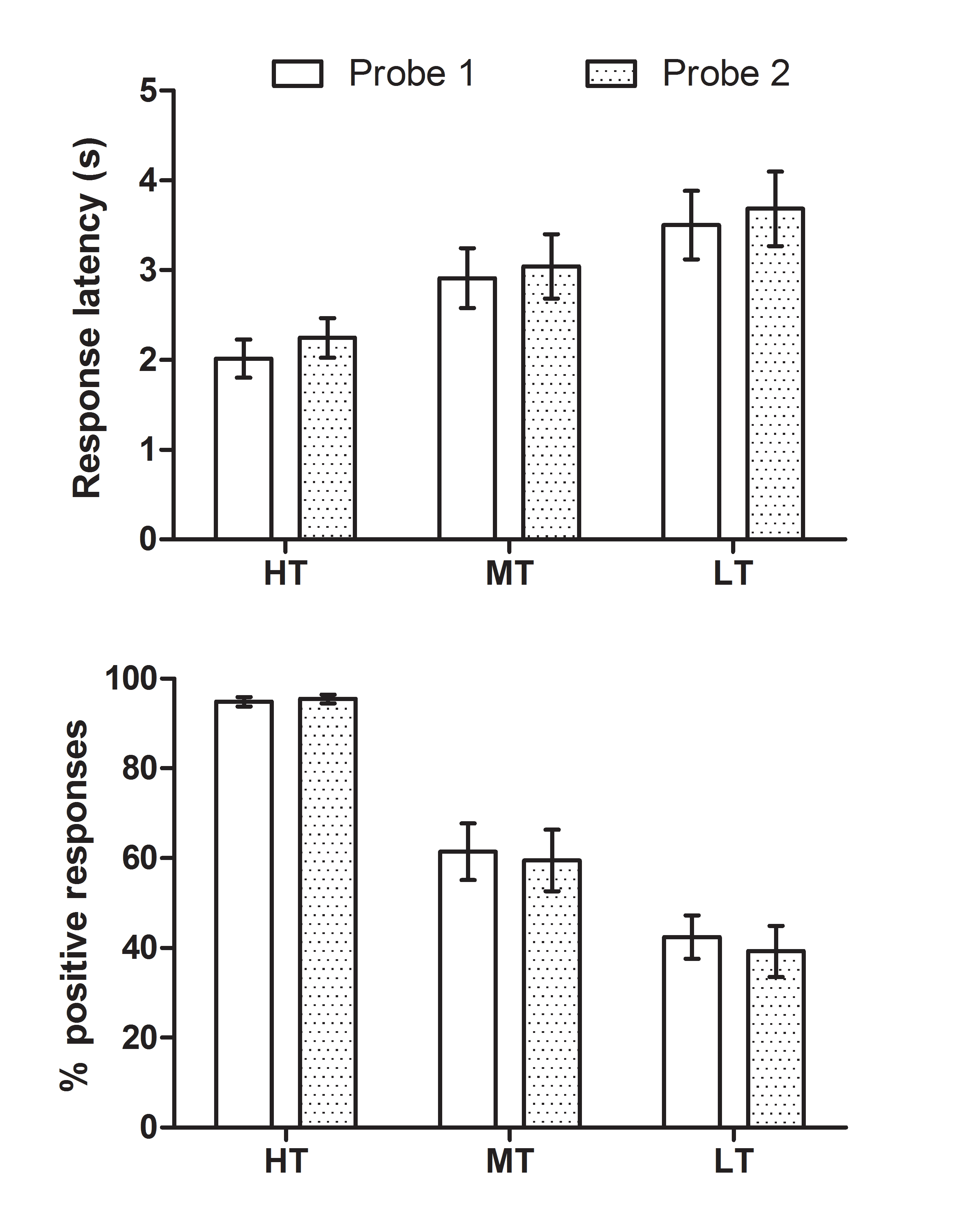

Supplement: S1 Fig — Two probe tests were conducted prior to experimental manipulations to use for diffusion model fitting and validation. (A) Latency to respond to each tone. (B) The percentage of positive responses made for each tone. Data represent mean ± SEM; n = 16. HT—high reward tone; MT—midpoint tone; LT—low reward tone. (TIF) [file pone.0152592.s001.tif]
