# Supplementary material for: Diffusion Modelling Reveals the Decision Making Processes Underlying Negative Judgement Bias in Rats
Source: PLoS One. 2016 Mar 29;11(3):e0152592. doi: 10.1371/journal.pone.0152592 (PMC4811525; doi:10.1371/journal.pone.0152592)
Supplement: S1 File — (DOCX) [file pone.0152592.s002.docx]

# **S1 File**

# **Supporting Materials and Methods**

## **Apparatus**

Operant chambers (30.5 x 24.1 x 21.0 cm) used for behavioural testing were housed inside a light-resistant and sound-attenuating box. They were equipped with two retractable response levers that were positioned on each side of the centrally located food magazine. The magazine was connected to the sugar pellet dispenser, and had a house light (28 V, 100 mA) located above it. An audio generator (ANL-926, Med Associates, Sandown Scientific, UK) produced tones that were delivered to each chamber via a speaker positioned above the left lever. Operant chambers and audio generators were controlled using K-Limbic software (Conclusive Solutions Ltd., UK). Tone, associated lever and associated outcome were fully counterbalanced across rats. The reference tones (2 and 8 kHz) were chosen so that the range they span is within an approximately linear part of the mean frequency difference limen in hooded rats [1], and matches tones used previously in a reward-punishment version of the task [2]. This meant the midpoint ambiguous probe tone could be equidistant from the reference tones. For all tones, loudness was adjusted to account for differences in absolute thresholds according to the hooded rat audiogram [3].

## **Behavioural task**

Training stages and criteria are detailed in S1 Table. Training sessions consisted of 100 trials or lasted a maximum of one hour. Unless otherwise specified in Table 1, response levers were extended at the beginning of every session and remained extended for the duration of the session. During sessions pressing the incorrect lever was punished by a 5 s timeout, as was an omission if the rat failed to press any lever during the 20 s tone. Tone presentations were separated by an inter-trial interval of 5 s, during which time premature responses on either lever were punished by a 20 s timeout. During a timeout, the house light was illuminated, and responses made on levers were recorded but had no programmed consequences.

## **Modelling**

Two probe tests carried out before experimental manipulations were used for model fit validation. Validation was carried out for all rats (*n* = 16). Multiple models were tested using different combinations of parameters that were fit to each tone individually rather than from data from all tones together to identify the parameter combination that produced best model fits. As described below and following recommendations given in Voss et al. [4], model fit was assessed using Kolmogorov-Smirnov (KS) test statistics output by fast-dm-30, along with graphical inspection of Q-Q plots. The KS test statistic is the maximum absolute vertical distance between the empirical and the predicted cumulative density functions (CDF) of the response time (RT) distributions. For multiple trials in a task, *n*, it is computed as:

$$KS = maxi = 1\ldots n\left| eCDF\left( RTi \right)-pCDF\left( RTi \right) \right|,$$

where *eCDF* and *pCDF* are the empirical and predicted CDFs, respectively, and *RTi* is the response latency in trial *i*. When some parameters are estimated for different experimental conditions (in this case for different tones), fast-dm-30 outputs a goodness of fit probability value which is the product of individual minimised KS test statistics for each condition. *p-*values < 0.05 indicate that the model does not demonstrate a good fit. For graphical model fit, RTs corresponding to the first (0.25), second (0.5) and third (0.75) quartiles taken from empirical (behavioural data) and predicted (model) CDFs were compared for each rat using scatterplots. Satisfactory model fit can be concluded if all the data points lie near the main diagonal [see 5 for an example].

The empirical and predicted percentage of positive responses were also compared by calculating the AuC for the high reward responses CDF.

During model fitting and modelling of experimental data, trials with response latencies shorter than 200 ms (mean ± SEM = 2.67 ± 0.20% of data points) were removed from the analysis. Previous studies [6-8] have excluded very rapid responses as these do not reflect a true decision making accumulation process in response to the cue, being instead considered as fast guesses [9]. This also considerably improves model fit.

# **References**

1. Syka J, Rybalko N, Brožek G, Jilek M. Auditory frequency and intensity discrimination in pigmented rats. Hearing Res. 1996; 100(1): 107-113.
2. Anderson M, Munafò M, Robinson EJ. Investigating the psychopharmacology of cognitive affective bias in rats using an affective tone discrimination task. Psychopharmacology. 2013; 226(3): 601-613. doi: 10.1007/s00213-012-2932-5.
3. Heffner HE, Heffner RS, Contos C, Ott T. Audiogram of the hooded Norway rat. Hearing Res. 1994; 73(2): 244-247.
4. Voss A, Voss J, Lerche V. Assessing Cognitive Processes with Diffusion Model Analyses: A Tutorial based on fast-dm-30. Front Psychol. 2015; 6: 336. doi: 10.3389/fpsyg.2015.00336.
5. Voss A, Rothermund K, Gast A, Wentura D. Cognitive processes in associative and categorical priming: a diffusion model analysis. J Exp Psychol Gen. 2013; 142(2): 536-559. doi: 10.1037/a0029459.
6. Ratcliff R, Rouder JN. Modeling response times for two-choice decisions. Psychol Sci. 1998; 9(5): 347-356.
7. Voss A, Rothermund K, Brandtstädter J. Interpreting ambiguous stimuli: Separating perceptual and judgmental biases. J Exp Soc Psychol. 2008; 44(4): 1048-1056. doi: http://dx.doi.org/10.1016/j.jesp.2007.10.009.
8. White CN, Ratcliff R, Starns JJ. Diffusion models of the flanker task: Discrete versus gradual attentional selection. Cogn Psychol. 2011; 63(4): 210-238. doi: http://dx.doi.org/10.1016/j.cogpsych.2011.08.001.
9. Vandekerckhove J, Tuerlinckx F. Fitting the Ratcliff diffusion model to experimental data. Psychon Bull Rev. 2007; 14(6): 1011-1026.
