# Supplementary material for: Diffusion Modelling Reveals the Decision Making Processes Underlying Negative Judgement Bias in Rats
Source: PLoS One. 2016 Mar 29;11(3):e0152592. doi: 10.1371/journal.pone.0152592 (PMC4811525; doi:10.1371/journal.pone.0152592)
Supplement: S1 Table — Summary of the stages used to train rats on the reward-reward operant ambiguous-cue interpretation task. The criteria column specifies the criteria rats were required to meet before they could advance to the next stage of training. Training time shows the maximum length of time necessary to meet criteria for each stage. Training time is given in both sessions and days, as some training stages were conducted twice per day. ITI–inter-trial interval. Between the end of training and the start of experimental manipulations another eight weeks elapsed which included the following: testing to provide data for diffusion model fitting and validation and to ensure stable responding to ambiguous tones during repeated probe tests over time (four weeks in total; data used for diffusion model fitting is shown in S1 Fig); a break with no testing during which rats received ad libitum food in the home cage (two weeks); and re-baseline sessions following the break (two weeks). (DOCX) [file pone.0152592.s003.docx]

# **S1 Table**

| **Stage** | **Description** | **Criteria** | **Training time** |
| --- | --- | --- | --- |
| **1 – Magazine**  **training** | Tone (high or low only on alternate sessions) played for 5 s followed by release of one food pellet into magazine; 30 s ITI. No levers available. | All sugar pellets eaten | 4 sessions  (4 days) |
| **2 – Lever**  **training** | Tone (either high or low presented pseudorandomly) plays for 20 s: response on either lever during tone rewarded with one food pellet; 10 s ITI. | > 70 responses over 2 consecutive sessions | 3 - 5 sessions  (3 -5 days) |
| **3 – Tone**  **training** | Tone (high or low only on alternate sessions) plays for 20 s: response on correct corresponding lever only rewarded with one food pellet; 5 s ITI. | > 70% accuracy and  < 1:1 ratio of correct:premature responses | Overall 26 sessions for both stages  (13 days).  4 - 12 tone training sessions, remainder discrimination training sessions |
| **4 – Discrimination**  **training** | Tone (either high or low presented pseudorandomly) plays for 20 s: response on correct corresponding lever only rewarded with one food pellet; 5 s ITI. | > 70% accuracy for both tones, no significant differences on analysed behavioural measures over three sessions and < 1:1 ratio of correct:premature responses |  |
| **5 – Reward**  **magnitude**  **training** | As Stage 4 but response on correct corresponding lever only rewarded with four food pellets for high reward tone and one food pellet for low reward tone; 5 s ITI. | As for Stage 4 but with > 60% accuracy for both tones  (to allow for biases in responding to reference tones caused by the difference in associated reward magnitude) | 8 sessions  (8 days) |
