# Supplementary material for: Diffusion Modelling Reveals the Decision Making Processes Underlying Negative Judgement Bias in Rats
Source: PLoS One. 2016 Mar 29;11(3):e0152592. doi: 10.1371/journal.pone.0152592 (PMC4811525; doi:10.1371/journal.pone.0152592)
Supplement: S2 Table — Data for number of premature responses made across the whole session (trials where a response was made in the 5 s ITI before tone presentation) and percentage of omissions for each tone (trials where no lever press occurred during 20 s tone presentation divided by total completed trials). These are data are separated into experiment and manipulation/group. * denotes significant difference (p<0.05) compared to control (repeated measures ANOVA and post-hoc test). All values are mean ± SEM. (DOCX) [file pone.0152592.s004.docx]

| **Experiment** | **Manipulation / Group** | | **Premature (#)** | **Omissions (%)** | | |
| --- | --- | --- | --- | --- | --- | --- |
|  |  |  |  | **High tone** | **Mid tone** | **Low tone** |
| **1** | Acute restraint stress | Control | 20.18 ± 3.13 | 0.00 ± 0.00 | 0.45 ± 0.30 | 0.45 ± 0.30 |
|  |  | Restraint stress | 25.64 ± 3.18 | 0.51 ± 0.35 | 0.57 ± 0.57 | 0.55 ± 0.55 |
|  | FG7142 | Vehicle | 28.40 ± 7.13 | 0.00 ± 0.00 | 0.00 ± 0.00 | 0.00 ± 0.00 |
|  |  | 3.0 mg/kg | 18.40 ± 2.71 | 0.25 ± 0.25 | 2.33 ± 1.19 | 2.93 ± 1.51 |
|  |  | 5.0 mg/kg | 21.90 ± 2.30 | 1.74 ± 0.77 | 3.15 ± 1.78 | 6.27 ± 1.92* |
| **2** | Control group | Pre-stress | 23.50 ± 4.43 | 0.00 ± 0.0 | 0.25 ± 0.25 | 0.25 ± 0.25 |
|  |  | Stress | 24.20 ± 4.38 | 0.00 ± 0.00 | 0.17 ± 0.17 | 0.37 ± 0.37 |
|  |  | Post-stress | 21.80 ± 5.77 | 0.00 ± 0.00 | 0.07 ± 0.07 | 0.13 ± 0.13 |
|  | RS&SI group | Pre-stress | 19.10 ± 4.98 | 0.00 ± 0.00 | 0.75 ± 0.50 | 0.25 ± 0.25 |
|  |  | Stress | 27.00 ± 6.38 | 0.41 ± 0.33 | 0.78 ± 0.52 | 1.04 ± 0.72 |
|  |  | Post-stress | 19.23 ± 7.39 | 0.03 ± 0.03 | 0.27 ± 0.21 | 0.40 ± 0.34 |

# S2 Table
