# Supplementary material for: Diffusion Modelling Reveals the Decision Making Processes Underlying Negative Judgement Bias in Rats
Source: PLoS One. 2016 Mar 29;11(3):e0152592. doi: 10.1371/journal.pone.0152592 (PMC4811525; doi:10.1371/journal.pone.0152592)
Supplement: S3 Table — The percentage of rapid responses that were removed from behavioural data before diffusion model analysis. Rapid responses were defined as responses occurring with a latency of less than 200 ms. All values are mean ± SEM. (DOCX) [file pone.0152592.s005.docx]

# S3 Table

| **Experiment** | **Manipulation / Group** | | **Rapid responses removed (%)** |
| --- | --- | --- | --- |
|  |  |  |  |
| **1** | Acute restraint stress | Control | 3.04 ± 0.80 |
|  |  | Restraint stress | 3.19 ± 0.98 |
|  | FG7142 | Vehicle | 2.88 ± 0.82 |
|  |  | 3.0 mg/kg | 1.81 ± 0.40 |
|  |  | 5.0 mg/kg | 1.78 ± 0.91 |
| **2** | Control group | Pre-stress | 2.92 ± 0.83 |
|  |  | Stress | 3.59 ± 0.83 |
|  |  | Post-stress | 2.98 ± 0.89 |
|  | RS&SI group | Pre-stress | 1.17 ± 0.51 |
|  |  | Stress | 1.75 ± 0.78 |
|  |  | Post-stress | 2.01 ± 1.03 |
