# Supplementary material for: Diffusion Modelling Reveals the Decision Making Processes Underlying Negative Judgement Bias in Rats
Source: PLoS One. 2016 Mar 29;11(3):e0152592. doi: 10.1371/journal.pone.0152592 (PMC4811525; doi:10.1371/journal.pone.0152592)
Supplement: S4 Table — Data for other parameters fit by the diffusion model across all tones: the across-trial variability in decision starting point (szr) the non-decision time (t0) and the difference between the two responses in speed of execution (d). These data are separated into experiment and manipulation/group. All values are mean ± SEM. (DOCX) [file pone.0152592.s006.docx]

| **Experiment** | **Manipulation / Group** | | **Diffusion model parameter** | | |
| --- | --- | --- | --- | --- | --- |
|  |  |  | ***szr*** | ***t_0_*** | ***d*** |
| **1** | Acute restraint stress | Control | 0.24 ± 0.03 | 0.41 ± 0.10 | 0.017 ± 0.010 |
|  |  | Restraint stress | 0.31 ± 0.05 | 0.37 ± 0.08 | 0.001 ± 0.029 |
|  | FG7142 | Vehicle | 0.31 ± 0.06 | 0.45 ± 0.10 | 0.003 ± 0.010 |
|  |  | 3.0 mg/kg | 0.30 ± 0.05 | 0.48 ± 0.12 | 0.005 ± 0.007 |
|  |  | 5.0 mg/kg | 0.33 ± 0.03 | 0.75 ± 0.20 | 0.022 ± 0.016 |
| **2** | Control group | Pre-stress | 0.35 ± 0.06 | 0.37 ± 0.10 | 0.026 ± 0.010 |
|  |  | Stress | 0.34 ± 0.05 | 0.26 ± 0.08 | 0.030 ± 0.005 |
|  |  | Post-stress | 0.36 ± 0.05 | 0.30 ± 0.09 | 0.006 ± 0.005 |
|  | RS&SI group | Pre-stress | 0.24 ± 0.03 | 0.48 ± 0.15 | 0.021 ± 0.005 |
|  |  | Stress | 0.26 ± 0.02 | 0.48 ± 0.14 | 0.025 ± 0.013 |
|  |  | Post-stress | 0.29 ± 0.03 | 0.53 ± 0.19 | 0.008 ± 0.009 |

# S4 Table
